# Editorial: Understanding Extreme Sports: A Psychological Perspective

**DOI:** 10.3389/fpsyg.2019.03029

**Published:** 2020-01-31

**Authors:** Eric Brymer, Francesco Feletti, Erik Monasterio, Robert Schweitzer

**Affiliations:** ^1^Australian College of Applied Psychology (ACAP), Sydney, NSW, Australia; ^2^Politecnico di Milano, Milan, Italy; ^3^Department of Psychological Medicine, Christchurch School of Medicine, University of Otago, Dunedin, New Zealand; ^4^The School of Psychology and Counselling, Queensland University of Technology, Brisbane, QLD, Australia

**Keywords:** extreme sports, well-being, learning, performance, motivations, definitions

A new class of sport has emerged in the last few decades, variously called extreme, adventure, action, and lifestyle sports. These activities are revolutionizing the notion of sport, exercise and physical activity and overtaking many traditional sports in terms of participation, and influence. They have developed into a significant worldwide phenomenon with considerable social and economic impact (Brymer and Schweitzer, 2017a). While participant numbers in many traditional team and individual sports such as golf, basketball, and racket sports have declined over the last decade or so, participant numbers in so-called extreme sports have surged. The current trajectory suggests that traditional sports will soon play second fiddle to these new and exciting opportunities. With the continually rising participation rates in these activities, science and medicine is starting to give these sports the same attention already given to traditional sports (Feletti et al., 2017). However, this attention needs to consider the unique and nuanced characteristics of the people involved, their motivation, and the activities. As already noted and further highlighted by many articles in this special edition, extreme sports are not well-served by approaches that stem from traditional sports research (Arijs et al.).

From a psychological viewpoint, while the tendency has been to assume that the activities and experiences described as extreme sports are somehow homogenous, research is questioning these assumptions and revealing important nuances between activities and subdisciplines within the same activity (Collins and Brymer, 2018). These nuances are pointing to understandings that not only help explore extreme sports more generally but also revealing important information about what it means to be human (Brymer and Schweitzer, 2017b).

Traditional explanations for why extreme sports have become so popular are varied but for the most part stem from a notion that extreme sport participation is deviant or undesirable. Indeed, there are many examples of the downside of extreme sports. For example, Everest and other high and popular mountains have long been associated with the rubbish left over by mountaineers (Bishop and Naumann, 1996) and with notorious images of mountaineers walking past dying colleagues (Elmes and Barry, 1999). This special edition does not set out to refute these happenings, rather add to the work already undertaken.

Traditional notions on motivations include perspectives that stress rebellion against a society that is becoming too risk-averse. For others, it is about the spectacle and the merchandise that is associated with organized activities and glamorous athletes. Authors have also proposed that extreme sport participation is merely an outlet for those people attracted by risk and danger or the desire to brag. For others still, it is about the desire to belong to sub-cultures and the glamor that goes with extreme sports. This confusing array of explanations is unfortunate as despite their popularity there are still negative perceptions about extreme sports participation particularly acute immediately after accidents or adverse incidents.

Attempts to categorize extreme sport activities have been challenging but as authors in this special edition attest (see Immonen et al.; Cohen et al. in this edition) we are rapidly moving into an age where the nuances make a considerable difference when we investigate motivations, performance and outcomes, and as such clarity is fast becoming essential. Traditional attempts to define extreme sports have variously focused on either task elements, environmental characteristics or individual participants, separately, or in combination. From a task perspective, the traditional emphasis has been on the activity being high-risk or dangerous with the potential to cause considerable harm or even death of the participant. More recently, the risk notion has been downplayed, and extreme sport activities have been differentiated from mainstream sports because extreme sports are typically not governed by external rules and regulations. In this special edition, activities have been described as both competitive (see Cohen et al.) or non-competitive and creative (see Immonen et al.; Cohen et al.).

The traditional participant perspective has generally accepted that participants are outliers (at times deviant), emphasizing personality, emotional impact, and the role of adrenaline and thrill. Participants are assumed to search out opportunities for participation because they have underlying personality structures that demand novel, risky, and dangerous experiences. The environment perspective has leant on both social and physical explanations. Those studies espousing the social environment most often write about the impact of social pressure on performance or how extreme sports are an invention stemming from sub-cultural desires to rebel against mainstream society or demonstrate perceived masculine characteristics. Those studies focusing on the physical environment tend to project the environment as key to understanding extreme sport participation and focus on the natural world as dynamic, unconstrained by human intervention, or dangerous and needing to be tamed or controlled. The traditional approach has suggested that participants compete against the environment. However, research has critiqued this perspective, pointing out that participants recognize competition with the environment would be fruitless. More recent research has differentiated extreme sport participation from traditional sport participation because the environment is not constrained by artificial boundaries (Feletti and Brymer, 2018).

Part of the confusion seems to be that extreme sports activities are continually evolving, partially facilitated by technology and partially by the creative capacities and skills of participants. For example, extreme activities such as BASE (an acronym for Buildings, Antennae, Span, Earth) jumping has evolved to include proximity flying. Tow-in surfing, developed in the 90s and facilitated the capacity to ride bigger waves. Equally, although extreme sports are still assumed to be a Western pastime, there has been considerable uptake across the globe (Brymer and Schweitzer, 2017a). The idea that extreme sports are only for the young is also changing as participation rates across generations are growing. Baby boomers are enthusiastic participants of a variety of adventurous sports more generally. The gender divide is another presupposition that is being questioned (see Frühauf et al.; Monasterio et al. in this edition). Extreme sports now support a multi-billion dollar industry, and the momentum seems to be intensifying. Interestingly, as noted above, this has also opened up opportunities for versions of extreme sports to be included in mainstream events such as the Olympic Games. For example, kitesurfing made its first appearance at the 2018 Youth Olympic Games in Buenos Aires, Argentina and will be included in the 2024 Paris Olympic Games. Surfing, rock climbing and skateboarding will also be making their debut at the 2020 Olympic Games in Tokyo, Japan. Media and the general public have been captivated by images of high velocity, high altitude, forceful accelerations, and extreme physical precision required to perform these sports. In turn, these developments have has added to the confusion about the importance of competition.

There is a pressing need for clarity. The dominant research perspective has focused on positivist theory-driven perspectives that attempt to match participation in extreme sports against predetermined characteristics. For the most part, empirical research has conformed to predetermined societal perspectives. Other ways of knowing have revealed more nuanced perspectives of the human dimension of extreme sport participation. There has been considerable development of research into extreme sports since the early days in the 1960s. Studies focusing on medicine, sociology, physiology, and psychology are now being published in mainstream outlets, and extreme sports research has become a visible part of many traditional research agendas. While, researchers are still working to understand the experience better map out how best to support learning and document the outcomes from extreme sport participation, researchers also realize that extreme sports have a lot to offer research in mainstream sports and findings from extreme sports research are proving pertinent to our understanding of everyday experiences.

The **impact of extreme sports on health and well-being** represents one of the most interesting themes to emerge from the extreme sport experience reported in this special edition and supported by contemporary research. Research in this special edition indicates that, if managed effectively (Schüler et al.; Buckley, in this edition), participation in extreme sports can induce positive emotions and resilience, and facilitate the development of skills and physical capacities that support flourishing in everyday life (see Maclntyre et al. and Hetland et al.). Maclntyre et al., highlight findings that support the idea that extreme sport participation can lead to positive relationships with the natural world and pro-environmental behaviors (Brymer et al., 2009; Brymer and Gray, 2010). Extreme sports are described as meaningful and life-enhancing (see Immonen et al.) with the potential to be used as therapeutic interventions to address everyday psychological issues and drug abuse (see Roberts et al.; Roderique-Davies et al.). Holmbom et al., argue that extreme sport participation is very different from traditional sports perhaps because the potential outcome is far more serious. They argue that extreme sports have profound positive transformational capacities and that the skills learnt during extreme sports were relevant for, and enhanced everyday life.

Another interesting theme to emerge from this special edition, and highlighted in the current discourse on extreme sports, concerns **how extreme sports should be defined**. Risk does not turn out to be as central a notion as has traditionally been assumed. Buckley points to a definition that combines person and task elements of participation. Extreme sports are placed further along a continuum from adventure sports where death is a potential; survival relies on “moment to moment skill” and emotions involved in thrill are the central notions. Cohen et al. propose extreme sports should be defined by the type of activity. They suggest that extreme sports are by nature competitive activities undertaken in natural contexts with atypical physical challenges and the potential of death as an outcome. On the contrary, Langseth and Salvesen focuses on the role of values and Immonen et al. argue for a more creative and meaning based definition where extreme sports and traditional sports differ precisely because competition is not emphasized in extreme sports. Attention is given to aesthetic criteria rather than traditional quantitative parameters (e.g., distance, time, score) when assessing performance. Like Buckley, Cohen et al. argues that risk is what differentiates extreme from traditional sports. However, as above the importance of risk is questioned by Immonen et al. who point out that the risk focus has important limitations and provide a more relational appreciation of extreme sports as emergent activities that afford opportunities for existential reflection and self-actualization. Again, they emphasize the relationship between performer and environment to argue their case.

The third outcome of this special edition links to **learning and performance in extreme sports**. First, the idea that only certain people undertake extreme sports, usually determined by the personality characteristics of the participant or underpinned by gender or the adrenaline junky explanation might not stand up to scrutiny (Collins et al.). Research reported in this special edition suggests that individual participant characteristics are broad and not easily captured by personality measures or gender (Monasterio et al.; Frühauf et al.). Second, in contrast to more traditional perspectives that assume skilled performance relies on innate individual characteristics, articles in this special edition point to other mechanisms. Arijs et al., argue that performance in extreme sports is very different from performance in traditional sports because of the seriousness and potential consequences of the activity. Instead of a traditional narrative that emphasizes winning, extreme sports accentuate exploration, discovery and a relational perspective linking profound knowledge of self and task and attunement to information in the environment as key to effective performance. Seifert et al. (this edition), in their skill acquisition focus, support a relational perspective, arguing that learning and performance are linked to a productive person-environment relationship and the development of more effective attunement to affordances (invitations for action) in the environment. Learning in extreme sports is about how well the performer perceives opportunities for action that combine personal characteristics with environmental characteristics. Collins et al. follow a skills training model demonstrating the ways in which knowledge of performance in emerging competitive versions of extreme sports extends traditional sporting models. Developing skills and expertise to perform effectively in extreme sports also facilitates opportunities to rethink learning in other sports, and more generally across learning environments.

**Motives and motivations for participation in extreme sports** emerged as a fourth theme in this special edition. The majority of articles in this edition have questioned the assumption that a particular type of person participates in extreme sports or that the desire for risk and danger underpinned participation. McEwan et al. (this edition) point out that traditional assumptions that emphasize homogeneity in extreme sport participation might be limited. Instead, they draw attention to differences in motivations and interests even within the same sport, in their case, mountain biking. Monasterio and Cloninger (this edition) examined personality structures of BASE jumpers and mountaineers; they determined that while personality measures did not suggest that extreme sports athletes were a homogenous group, there were trends toward being controlled and adventurous. Men and women shared similar characteristics and constructs such as self-actualization were more critical in understanding the motivation of extreme sports people compared to the traditional notion of social pressure. Happiness, challenging oneself, being in nature, friendships, and balance were also noted as important factors (Hetland et al.; Frühauf et al.).

The themes highlighted above suggest four interesting take-home messages from this special edition:

1. Engaging in extreme sports and adventure more generally provides us with important insights into what it means to be human. From a practice and policy perspective, the scholarly study of extreme sport participation suggests that we should be finding ways to encourage participation across the lifespan and finding ways to include extreme sports and adventure in activities designed to enhance health, education and physical activity participation. While traditional perspectives on the reason for encouraging adventure may no longer be relevant, the impacts of participation in adventure are more essential today than ever before.
2. Extreme sports are complex. Looking for simple answers that emphasise aspects such as risk, personality, or social forces misses the point entirely. Perhaps more than any other type of human experience extreme sports provide a means to rethink the human-environment relationship and the importance of activities that do not focus on winning, rules, regulations, and manicured playgrounds. Extreme sports research should be encouraged across multiple disciplines.
3. Extreme sports participation is more likely to be suitable for the general population than not. Indeed, participation in extreme sports and their less extreme cousins support physical, social and psychological health and well-being across the lifespan and, notably, also the well-being of the planet. While there are challenges when it comes to the development of technology that supports advancements, in general, extreme sports should be encouraged, and should be considered significant positive activities worthy of consideration in policy development across the health, education, sport, and environment sectors. Extreme sports should be considered a fruitful approach for involving people in health-enhancing and active lifestyles. Young people, in particular, should be encouraged to take part in these activities to foster social interaction, informal learning, and socio-cultural integration.
4. Intriguingly, research reported in this special edition points to knowledge gained in extreme sports as useful for performance in other domains of life, such as business or sport.

In conclusion, extreme sports should not be considered activities for the few but fundamental aspects of human expression and the development of healthy populations and environments.

## Author Contributions

All authors listed have made a substantial, direct and intellectual contribution to the work, and approved it for publication.

### Conflict of Interest

The authors declare that the research was conducted in the absence of any commercial or financial relationships that could be construed as a potential conflict of interest.
